# To Harlestonensis

**Published:** 1808-08

**Authors:** 


### To HARLESTONENSIS.

Sir,

It is generally acknowledged, I believe, that there are offences, without the pale of the law, of greater magnitude, than many which are severely punishable by our criminal code. To personate another for the purpose of any pecuniary advantage, is made felony without benefit of clergy; but to assume the name of a respectable man, with the design of vilifying or degrading him, may be done with impunity; though the crime against society in the latter instance, is as much more serious than the former, as the value of character is to be estimated beyond the price of wealth. This preamble leads to a charge of a late attempt in the Med. Journal, to 'laugh to scorn' a very worthy gentleman;

Note by M. Thenard.

gentleman; and if, as some suspect, you are not the identical personage W. G. M D. intended to represent Dr. Girdlestone of Yarmouth, I shall hope for your assistance to strip off the lion's skin, and expose to the just indignation of the public, the degraded author of so vile an artifice; indeed, if you are not concerned in this fabrication, it will be incumbent upon you to aid the discovery by every possible elucidatiou; for at some future time, Harlestonensis may, in propria persona, become the butt of the slanderer, when we find, as in this case, how easily it is effected, that no talents are requisite but those for defamation.

Your *first* letter contains so little worthy of observation, and is so free from all offensive language, that our astonishment is excited at the angry and supercilious reply of Dr. G.—*This supposed answer*, produces your *second* letter, which could only be justified by a firm persuasion in your own mind that it was really written by that physician.

Now, Sir, I would ask, did you not know before the second letter was printed, that W. G. M. D. was a fictitious person?

Were you not informed *(in time to have suppressed this unprovoked attack upon Dr. G.)* that he, (Dr. G.) was not your correspondent?

If you do not give a plain and satisfactory answer to these queries, your credit must suffer in the judgment of every honest man.

VERITAS.

*July* 10, 1808.

